# HOW HAS NARRATIVE BEEN USED IN GERONTOLOGY?: FINDINGS FROM A QUALITATIVE EVIDENCE SYNTHESIS

**DOI:** 10.1093/geroni/igac059.960

**Published:** 2022-12-20

**Authors:** Kate de Medeiros

**Affiliations:** Miami University, Oxford, Ohio, United States

## Abstract

Narrative gerontology emerged as a concept in the 1990s to describe the ways that people age biographical as well as biologically. Since then, narratives and narrative approaches have gained popularity as methods and ways of knowing. This paper presents findings from a qualitative evidence synthesis (QES) of narrative within the gerontological literature. ). QES describes methods to systematically review qualitative evidence (e.g., extracted text). The purpose is to gain a deeper understanding of a concept rather than evaluate study designs or findings. An initial search returned 1,480 papers. Upon further analysis, the following three large categories with regards to narrative were identified: definitions, accounts, processes, and possibilities. Overall, findings provide an comprehensive overview of uses of narrative within gerontology.

